# The wolf, the lamb, and the big “Oh!”: voids, (w)holes, and epitaphic emptiness in Frances Burney’s *Hubert de Vere*

**DOI:** 10.12688/openreseurope.16439.1

**Published:** 2023-09-06

**Authors:** Francesca Saggini

**Affiliations:** 1 The School of Literatures, Languages & Cultures, The University of Edinburgh, Edinburgh, Scotland, EH8 9JU, UK; 2 DISTU, Universita degli Studi della Tuscia, Viterbo, Lazio, 01100, Italy

**Keywords:** Frances Burney; Romantic drama; Tropology; Greek Tragedy; Historical drama; Isle of Wight; Pastoral; Performance

## Abstract

This essay explores the character of Cerulia in Frances Burney’s dramatic play, *Hubert de Vere*, composed and revised in the 1790s, yet never published or staged in Burney’s lifetime.

Cerulia seems to eschew any easy dramatic categorization, as she cannot be identified with the heroine of the play. Undeniably, she is a victim, but of whom/what, we may wonder? Does attempting to define the nature of the *hamartia* of which Cerulia remains victim lead the “ideal” reader/viewer toward either fate/the gods or, rather, social apparatuses? And, finally, what about the eponymous protagonist Hubert de Vere? Is it correct to identify de Vere as the actant “hero”, or perhaps as per the sub-category “villain hero” so popular in late eighteenth-century dramas?

Burney’s adroit exploitation of tropology and literary allusion in *Hubert de Vere* will be at the centre of this essay. In particular, I will examine the last act of the play, where the themes of confinement, imprisonment, and escape take on tragic hues. Though unpublished until 1995, these scenes are among the most vivid and, indeed, the most shocking Burney ever wrote. It is my contention that a long overdue appraisal of female characterisation in *Hubert de Vere* can shed novel light –at once both disturbing and liberating– on Frances Burney’s oeuvre at large.

## Plain Language summary

This essay explores the character of Cerulia in Frances Burney’s play *Hubert de Vere*, composed and revised in the 1790s, yet never published or staged in Burney’s lifetime.

Cerulia seems to eschew any easy dramatic categorization. Undeniably, Cerulia is a victim, but of whom/what? Can we consider her the heroine of the play? And what about the leading male character, Hubert de Vere? Is it correct to identify de Vere with the character of the “hero”?

Burney’s skilful exploitation of rhetorical figures and literary allusion in *Hubert de Vere* will be at the centre of this essay. In particular, I will examine the last act of the play, where the themes of confinement, imprisonment, and escape take on tragic hues. Though unpublished until 1995, these scenes are among the most vivid and, indeed, the most shocking Burney ever wrote. It is my contention that a long overdue appraisal of female characters in *Hubert de Vere* can shed novel light –at once both disturbing and liberating -- on Frances Burney’s oeuvre at large.

In the so-called traditional family, feelings are bound to roles, whereas it is exactly the opposite in the queer family: roles are masks that feelings wear when and if they are needed, otherwise better never.
                                                      Michela Murgia, “Queering the family”, Instagram post, 12 May 2023 ^1^

## Introduction. Conjuring Frances Burney’s ghost stage

A standalone essay can hardly accommodate the in-depth analysis needed to chart the complexities and, indeed, the novelties linking Frances Burney to the Romantic stage, a wide-ranging domain of investigation that I have explored in my two-year multimedia Marie Skłodowska Curie Action Project, *Opening Romanticism: Reimagining Romantic Drama for New Audiences*. Not only would this issue require a comprehensive explanation, but also systematic clarification, to defend the otherwise apparently unjustified inclusion of an author considered as predominantly belonging to the eighteenth-century novelistic tradition within a genre (the drama) and a canon (Romanticism) in the surface hardly akin to her. My case will therefore be made on a single, highly representative, item of inquiry, Burney’s long-forgotten ‘historical-gothic-pastoral’ play *Hubert de Vere*. ^2^ It is my hope that these prolegomena will encourage further critical analysis and reveal the interpretative possibilities inherent in a dramatic corpus, such as Frances Burney’s, mostly unfamiliar to her present-day readers as well as neglected by critics and ignored (if not openly mistreated) by coeval audiences.

By the mid-1780s, Burney had amassed substantial cultural and celebrity capital. ^3^ Her debut novel *Evelina, or the History of a Young Lady's Entrance into the World* (1778) had achieved indisputable success; her following novel, *Cecilia; Or, Memoirs of an Heiress* (1782), had confirmed that, indeed, she was no one-hit wonder and had made her by far the best-selling female author of the 1780s, translated into several European languages, including French. However, Burney’s situation changed completely only a few years later when her astonishing fame and solid reputation won her an unexpected, supposedly life-long royal appointment. ^4^ Events then took a traumatic turn. Her new, highly coveted position as Second Keeper of the Queen’s Robes at the Court of George III (1786–1791), with its inflexible schedule and burdensome royal ceremonial, rapidly sapped her creativity and physically exhausted her, producing a state of prostration increasingly worsened by the distressing mental illness of the King (approx. 1788–89). It is precisely from October 1788 that, sequestered in a royal household besieged on all sides by growing apprehension for the health of the King (hence, of the nation at large) and surrounded by universal woe, Burney’s letters record the composition of a small number of playtexts which were neither performed nor published in the author’s lifetime, remaining in manuscript until 1995. ^5^ In the first of such letters, Burney records:

We are to stay here [Kew] some time longer […]; and so, in mere desperation for employment, I have just begun a tragedy. We are now in so spiritless a situation that my mind would bend to nothing less sad, even in fiction. ^6^

In October 1791, finally released from her draining Court responsibilities, Burney takes up the topic again in a letter jointly addressed to her sister Susanna Phillips and their friend, Mrs Locke: “I have been going on with my *third* Tragedy [a re-draft of *The Siege of Pevensey*]. I have two written, but never yet have had the opportunity to read them [.]” Crucially, the letter also provides a unique meta-dramatic commentary on Burney’s compositional style in the lines that follow: “[…] which, of course, prevents their being corrected to the best of my power, & fitted for the perusal of less indulgent Eyes—or, rather, of Eyes less prejudiced. Believe me, my dear friends, in the present composed & happy state of my mind, I could never have suggested these Tales of Woe;—but having only to connect, combine, contract, & finish, I will not leave them undone.” ^7^ The four Court plays Burney eventually left to us in different stages of completion – but for which, tantalisingly, she does not provide a title to her correspondents– are the dramatic fragment conventionally known as *Elberta*, and three manuscript plays, entitled *Edwy and Elgiva, Hubert de Vere*, and *The Siege of Pevensey*, copied out in beautiful handwriting in ordered booklets, suitable for private circulation, if perhaps not publication.

The long-delayed publication of these plays has kept critics away, apart from a very few, notably Joyce Hemlow, Margaret Anne Doody, Ellen Donkin, Barbara Darby, and Jaqueline Pearson. Even for the handful of scholars who have dealt with them, however, these texts have modest dramatic qualities, though perhaps they are not completely ‘wretched’, as they were often called by their contemporary reviewers. Arguably, to the modern reader and in the form in which they have come to us, they seem too long to be staged, characterizations are stereotyped, the endings are weak, and the plots convoluted and inconsistent. The style, rhetorical and emphatic, makes them sound clumsy and heavy to the modern ear. ^8^

It is my contention that the quartet of dramas that Burney wrote during her melancholic period at Court prompts our critical thinking in terms of such present-day practices and policies concerning gender relations, body politics, and agency. To me, these long-neglected plays represent an excellent sounding board to test the edifices of Burney’s poetics, to follow the fault lines of her compositional practice and trace its forms and models. In this regard, *Hubert de Vere*, probably the second of the Court dramas, may offer a particularly significant contribution to the study of Burney’s macrotext and, hopefully, the Romantic stage at large.

Moving from these introductory considerations, I have chosen to focus my analysis on a select number of research questions, each of which will hopefully contribute a *tessera* to the mosaic I aim to reconstruct in the following four sections of the essay. When was *Hubert de Vere* probably composed and possibly revised? And have this timeline and history of the play’s composition and early revisions any significance? As far as the chronotope of the drama is concerned, what might have been the significance of the chronotope of the plot, its where-when – *i.e.,* the Isle of Wight and the twelfth century – for Burney and the contemporary audience? Why is one of the two main female characters called Cerulia? How does this naming technique, and tropology at large, contribute to Frances Burney’s strategy of dramatic characterization, hence to the overall ideologic agenda of the play? Finally, what can the study of a text such as *Hubert de Vere*, which remained unstaged and unpublished, teach us about Burney's poetics, her inter-theatrical and inter-cultural influences, and her creative routes?

## *Hubert de Vere* and Burney’s stage will

I understand *Hubert de Vere* as a true imaginative crucible for Burney, a kind of creative gymnasium – or a rehearsal area, if we want to remain in the theatrical sphere – in which to re-propose and revise models and forms that came to shape the drama, both overtly and covertly, over the many decades in which the play text was revised. ^9^ Moving from the inevitable need to approach Burney across the several decades and the various genres in which she operated, which made her a notably “protean” writer, ^10^ here I want to juxtapose two cross-temporal perceptions of Frances Burney, the multifaceted, Janus-like author. The first is a coeval statement made by a Burney family friend, Samuel Crisp, one of her many diligent (possibly too diligent) literary advisors. The second comes from the critic Claire Harman, a modern-day biographer of Burney.

On 25 January 1782, just about six months before the publication of Burney’s second novel, the astonishingly successful *Cecilia*, Samuel Crisp wrote to his sister Sophia, later Mrs Gast:

You see how triumphantly [Fanny] goes on. If she can coin gold at such a Rate, as to sit by *a warm Fire*, and in 3 or 4 months … gain £250 by scribbling the Inventions of her own Brain – only putting down in black and white whatever comes into her own head, without labour drawing simply from her own Fountain, she need not want money. ^11^

Crisp’s somewhat cavalier judgement dismisses Burney as a kind of unwitting goose that laid the (novelistic) golden eggs, protected by domestic intimacy – significantly, Crisp mentions a warm fire, a synecdoche for cosy unprofessionalism to which I will return presently – a young, sheltered female scribbler happily oblivious of the merciless demands enjoined on the (implicitly male) professional author. The construction of genteel and ladylike amateurism evoked by Crisp, however, clashes with what was instead affirmed, many years and many critical revisions later, by Claire Harman, who expresses an equally definite, though opposite, judgement. In her discussion of Burney’s post- *Cecilia* works, Harman pithily states: “Fanny Burney knew her market.” ^12^ This rather apodictic assertion refers in particular to the literary and editorial choices made by Burney in the 1790s and is based, I feel, on good evidence.

By mid-1794, after her marriage to Alexandre d’Arblay, an impecunious French officer who had emigrated to England, and several months pregnant, Burney must have realized that the economic needs of her new family made it imperative that she find a secure and immediate source of income. And, interestingly, it was on the Court dramas that Burney initially pinned her hopes. Although her letters never indicate any title, we assume that Burney gave a manuscript copy of *Hubert de Vere* to her self-appointed literary agent, her brother Charles Burney jr. In an interesting example of green room homo-social networking, Charles would have had the possibility to submit the draft manuscript to his drinking buddy John Philip Kemble, the hardly impeccable acting manager of the Royal Theatre, Drury Lane, to then reach Richard B. Sheridan, the even less impeccable owner of the same theatre.

We are unsure whether *Hubert the Vere* ever made it to Sheridan. In 1797, Burney writes that in the past Kemble had accepted “a certain melancholy ditty in nine acts” for representation, but no date of acceptance is ever given, here or elsewhere in her epistolary: she simply writes “once.” ^13^ Whichever this unspecified “ditty” may have been, it is a fact that in the latter months of 1794, still eager for immediate financial profit, Burney chose to push forward for representation a second, more markedly historical play, *Edwy and Elgiva*, probably the earliest of her Court dramas. Indeed, *Edwy and Elgiva* was staged at Drury Lane Theatre on 21 March 1795, although for one night only, with unanimous negative reviews from the public and critics. Sadly, an income generator it was not destined to be, and Burney, somewhat reluctantly, set it aside for the time being.

Therefore, in hindsight, who is the more perceptive interpreter, Samuel Crisp or Claire Harman? Was Burney a gifted, yet inexperienced author, an amateur in the contemporary ruthless literary market (as in Crisp’s lady-like idealization) or, conversely, was she a discerning professional, well-versed in the ways of the trade, with a keen interest in the profitable stage business too? In support of Harman’s hypothesis, we may recall how savvily Burney drove forward the best deal for *Camilla, or a Picture of Youth* (1796), aware that her authorship of the previous two anonymous novels was an ill-concealed secret, and instructing that the proposals for printing the new novel by subscription appear forthwith, “if you [here she is writing to her father] think my name *still may stand*”? ^14^ Harman’s hypothesis seems also confirmed by a letter dated 13 June 1795 (remarkably, Burney’s birthday). This crucial epistle was addressed to her father, Dr Charles Burney, the literary whisperer who cast a life-long shadow on Burney's works – both published and never-to-be-published. Here, referring to *Camilla*'s publication plan, Frances Burney employs, almost defiantly, four times that all-important word: *business*, in an unambiguous declaration of professionalism, which well encapsulates her mind-set and the imperatives behind it.

All our deliberations made, even after your *discouraging* calculations, we still mean to hazard the subscription, as we *cannot* follow your hint to settle a *round sum* now received on our little *Nonpareil*—we shall require immediate service from whatever is the interest money—we only desire or mean to guard him the principal. I am sure you will feel this answer to be as equitable & satisfactory as it is sincere. We are obliged to keep a servant for him, which so much enlarges our expences, that an increase of our income, is become absolutely indispensable. And, indeed, for such casualities, I had previously determined, when I *changed my state*, to set aside all my innate & original abhorrences, & regard & use as resources myself, what had always been considered as such by others. ^15^

Now a wife, a mother and, above all, the only dependable breadwinner in her financially-strained family, Burney seems to finally feel liberated both in the sight of the World and, above all, her father, free at last “to dance Nancy Dawson with *no* Fetters on – there is the difference.” (Here I paraphrase the notorious anti-theatrical injunction addressed to her by Crisp on 19 January 1779, at the time of the familial suppression of Burney’s first play, *The Witlings*, never published or staged until the 1990s *.*) ^16^

Paradoxical as it may seem, it is my contention that the critically-neglected, never-acted, unpublished *Hubert de Vere* is an excellent testing ground to decide where lies the more truth, whether in Crisp’s version – the tale of the domestic and unpretentious goose that laid novelistic golden eggs in the cusp of the late 1770s and the early 1780s – or in Harman’s narrative of the savvy, though not uniformly successful, professional woman writer. A brief synopsis of *Hubert de Vere* is necessary at this point.

King John has exiled Hubert de Vere to the Isle of Wight on the charge of being a traitor. The culprit in this plot is Baron de Mowbray, who has orchestrated a dark persecution against de Vere. De Vere is in love with the noble Geralda, who has been tricked into marrying another man to save her uncle de Mowbray from ruin; though betrayed and exploited by her relative, of whom she is the helpless pawn, Geralda is considered false and wanton by De Vere. Taking advantage of de Vere’s misperception of Geralda, the Baron urges the naïve maiden Cerulia to fall in love with de Vere. Duped once again, De Vere is about to capitulate to the girl’s pure love when the sudden arrival of the vindicated Geralda, now widowed, obliges him to choose between the two women. Geralda is the chosen one; Cerulia goes stark raving mad and dies.

In the tragic climax of the play, De Vere's forgiveness, granted by King John upon eventually realizing the Baron's treachery, is followed by the discovery that the Baron is also Cerulia’s father. But in this woeful dénouement all is too late and to no avail for the unfortunate maiden. Geralda and De Vere can only desperately lament over her grave, in the hope that her innocence will be rewarded with eternal rest.

As I mentioned, *Hubert de Vere* was never represented, nor was it ever printed during Burney’s lifetime, though at some stage Burney thought to revise it and perhaps publish it “to make it readable for a fire-side,” as she writes to her father on 26 January 1797. ^17^ It is fascinating to see how prudently Burney manages to re-code, to the reassurance of her anxious parent, the female novelist’s domestic “warm fire” conjured by Crisp into the semi-public “fire-side” associated with closet drama or amateur familial readings. Likewise, it is worth noting that when Burney started racking her brains about how to make her dwindling cultural capital profitable, it is to *Hubert de Vere*, rather than her other skeleton drama, *Edwy and Elgiva*, that she originally turned in Summer 1794. This brings me back to the second research question I wish to address here: why did she choose to set her play in the twelfth-century Isle of Wight? Why did Burney think that this place/time might have been of interest to contemporary audiences, and how does this choice fit in her aesthetic agenda?

## The dramatic chronotope of *Hubert the Vere*

In response to the above queries, I could put forward this, only apparently, oxymoronic opinion: *Hubert de Vere* is a “modern ancient piece.” ^18^ I contend that the dramatic chronotope of the Isle of Wight during the reign of King John (1199–1216) must be analysed from the past-present point of view (what I call the interaxial nexus) in order to address the aesthetic challenges I mentioned in the previous section of this essay.

At the time of Burney’s composition/first revisions (on what I call the axis of the present), the Isle of Wight was daily in the news, as the privileged base for the naval operations in which the English fleet was deployed against the French fleet. This sustained media presence was accompanied by a growing interest in the island, also known as the Garden of England, the much-loved destination of a domestic tourism increasingly eager to visit national sites of significant historical and scenic interest. From a brief survey I made, I was able to retrieve at least eight tourist guides published in the 1790s describing the natural beauty of the island, many of them richly illustrated. ^19^ Burney herself had visited the Isle of Wight in the company of Hester and Henry Thrale during their tour of southern England in 1780, when they had had to leave Bath quickly to flee the Gordon Riots. The letter to Dr Charles Burney, dated 14 June 1780, detailing this part of their tour is disappointingly short, though the mention of a forthcoming trip to the Isle of Wight is unequivocal: “Adieu, dearest Sir,—I am half ashamed, now all is so safe, of troubling you so often; especially as I can have no answers,—I therefore shall now write only every *other* Day till I get to Brighton.—We go to Spithead to-morrow,—& thence to the Isle of Wight.” ^20^

The Isle of Wight merged picturesque beauty with the Sublime, alternating views of verdant inland pastures with the magnificent and awful Needles and the Freshwater Cave. *Hubert de Vere*’s stage directions, both direct and implicit, summarily evoke this aesthetic juxtaposition. Here “a view of the Sea” (I, p. 97) is placed alongside “a Rural Scenery, with distant Hills” (II, p. 106); elsewhere “a Country Village” (III, p. 133) gives way to tall “Cliffs” set against “the azure Sky” (II, p. 115). Deixis and dialogue conjure other evocative scenarios, exploiting the whole breadth and depth of the newly rebuilt, enormous Drury Lane stage: a good example is provided by the implicit stage directions “towards the summit of yon craggy hill” (II, p. 110) and “Through yon trees | Seest thou not [Cerulia’s cottage’s] green thatch” (IV, p. 138). By contrast, the most recurrent architectural signs in contemporary drama are absent. This lack is even more notable if we consider that the Drury Lane scenographer was the highly skilled William Capon (active in that theatre between 1794 and 1802), who also authored the magnificently gothic scenery in *Edwy and Elgiva*. Not even the “most terrible” church with “mouldering windows” in Act V (p. 149), a promising sign of the architectural Gothic on which Capon could have given full vent to his skills, finds scenic actualisation, appearing only in Cerulia’s account of her dreadful vision in the churchyard.

The Isle of Wight’s natural beauty justified its comparison with a modern-day English version of the classical *Arcadia felix*, and the *Hubert De Vere* dramatic personae comprise bucolic characters such as three shepherdesses and a herdsman. The wealth of monuments and ancient buildings dotting the island recall a complementary historical dimension, once again essential to Burney's poetic weaving (here, we move on what I call the axis of the past). In 1215 King John, a character who had already acquired a distinct literary *persona* by the late eighteenth century, was obliged to confirm the liberties and privileges granted by King Henry II to the discontented English barons in a document that became known as Magna Carta, the Great Charter. Subsequently, King John retired to the Isle of Wight for three months, until the Pope annulled the Charter. ^21^

Though elitist in origin, as it meant to preserve ancient feudal privileges (including the privileges and inheritance of such noble widows as Burney’s Geralda, who is introduced in her role of *femme coverte* as “Glanville’s widow”, II, p. 107), during the eighteenth-century Magna Carta came to be considered a symbolic document in English political and social history and the wider British world. Extolled as the safeguard of individual liberties and equality, as well as a defence against the arbitrariness and oppression of royal absolutism, the eighteenth-century interpretation of the Charter recognised it as a fundamental contract designed to preserve England’s ancient constitution and to re-establish the rule of law and the right to equal justice. It was no coincidence that the American colonies appealed to Magna Carta when proclaiming their independence, ^22^ a catastrophic loss and a life-long source of pain for George III. More recently, inspiration was drawn from the Charter to emphasise the primacy of English law over the bloody Jacobin arbitrariness into which the French Revolution had fallen, as in the satirical print *The Contrast, 1793: British Liberty, French Liberty, Which is best?* by Thomas Rowlandson (1783), printed in the aftermath of the September Massacres and the arrest of King Louis XVI of France. ^23^ A final element in the interaxial past-present diptych imagined by Burney would not go unnoticed, at least at the time of the submission of the manuscript to Kemble in 1794. Since 24 May 1794 the Habeas Corpus Act had been suspended, conferring enormous power on William Pitt’s ministers and exacerbating the government’s repressive politics, particularly as concerned the definition of treason. ^24^

It is thus that, in the imaginary space-time of her drama, Burney manages to weld together the time and place of English History with those of her para-pastoral story. To borrow an influential contemporary argument, the personal is very often political in Burney: in her symbolic vocabulary, the macro-dimension of History (King John, the Baronial discontent, perhaps even the aristocratic widows' right to choose a new husband, as per Clauses seven and eight) finds actualisation in the micro-dimension of family life. This strategy was typical of much female literary production at the end of the century, both on the page (and I am thinking of the Gothic), and on the stage (and I am thinking of historical or semi-historical dramas).

Themes, form, and aesthetics confirm the long-dormant *Hubert de Vere* to be part of the Romantic dramatic tradition, the offspring of a quite recognisable epistemic and aesthetic configuration. On the ideological level, for instance, in *Hubert de Vere*, the illusion of a more equal society is dramatically upturned into a demagogic male-only – or at least an elitist, noblemen-only – utopia. The love/honour antithesis underlying Restoration heroic drama is thus recodified as the high/huble juxtaposition in Burney’s 1790s drama. ^25^ The fallacious and tragically misleading equation of nobility of caste (aristocracy) with nobility of soul (nobleness) is represented in the following couplet:

CERULIA No, Hubert, no! — Great, *noble* as thou art,
  And poor, and *humble*, and forlorn as I am […].
                                                        (II, p. 118; the emphasis is mine)

I find it bitterly ironic that standing at the very centre of this tragic plot is the virginal village maiden Cerulia, the blameless victim of social apparatuses *sub specie* the machinations of the father (the family dimension) and immemorial conventions and norms (the social, class and gender dimension). Consequently, the ideal of the Great Charter, a source of national pride, is challenged as false when placed alongside the reality and consequences of the dominant ideology. The actual effects are female suffering and the oppression of the weakest members of society, a persecution which Burney plays out in the arena of the family, or rather, of family politics.

## Strategies of a-naming: gender and family in *Hubert de Vere*

If King John’s Isle of Wight is central in Burney’s discourse, contemporary gender issues and the structures of gendered power are just as vital. And this consideration makes me close in on the tragic final ensemble in the last act of the play, set against the backdrop of a “Country Church Yard” (from the stage directions opening Act V, p. 146). As I argue in the final section of this essay, this subtle inter-cultural reference, harking back to Thomas Gray’s *Elegy Written in a Country Churchyard* (1751), confirms how inferential networks are deftly put at the service of aesthetics and ideology by Burney. Indeed, both explicit and indirect intertextuality informs, almost ‘directs’, the play at both the level of form and content.

*Hubert de Vere* is built on and around the dependence, interdependence and independence that connect four characters, the principal ones by virtue of the number of lines they are allocated. Here, I must specify that I prefer the neutral term ‘main characters’, rather than ‘protagonists’ as the former refers, on a purely quantitative level, to the distribution of lines, rather than to the characters’ more controversial dramatic functions. These four personages are Hubert De Vere; the Baron De Mowbray; Geralda, Glanville’s widow and De Mowbray’s niece; and the orphan Cerulia.

The questions of chronology and context have already been touched on in the previous section of this essay. It will now be best to proceed by closing up on Cerulia, a “hapless orphan” (II, p. 109), the foster daughter of the shepherd Eustace.

CERULIA. […] An unknown Orphan,
  Supported, barely, by some hidden bounty;
    Dear to no Father; by no Mother own'd;
    And stranger to all kindred benediction.
DE MOWBRAY. Oh torture!
                                                    (II, p. 112)

In a reprise of the high/humble pseudo-heroic antithesis I mentioned above, the proper name *Eustace* merges two different onomastic traditions into a single character: on the one hand Εὔσταχυς ( *Eústachys*), meaning “fruitful”, “fecund”, on the other Εὐστάθιος ( *Eustáthios*), meaning “steadfast”, “constant”. Both onomastic traditions converge on the character of the old shepherd who, significantly, is already dead before the play begins.

Like De Vere, Cerulia too was cast out on the island by the machinations of her aristocratic father, Baron De Mowbray. Eustace, Cerulia’s simple foster father, is the only male kin and the loving spirit who protected the girl, whose youth he watched over. However, he kept the secret of her identity, probably with a view to guard her. Though oppressed by remorse, De Mowbray remains unrepentant, apparently a victim of the forces of fate, but on closer inspection only of his obstinate blindness.

DE MOWBRAY […] Ye deep and awful thoughts! -- torment no longer
  With wearing, wasting, -- puerile retrospection.
                                                        (II, p. 112)

The final revelation of De Mowbray’s unowned paternity confirms *Hubert de Vere* as a drama of epistemic asymmetry, placing it firmly in the tradition of tragedy. As a result, De Mowbray’s belated acknowledgement is key: “Thy murderer is – thy Father! –", he cries, almost distracted (V, p. 162). Thus, the abortive anagnorisis between De Mowbray and Cerulia, consummated too late over the corpse of the daughter, confirms the paternal and, more broadly, the contextual crime already hinted at by the etymology of *Eustace*, the cruelly ironic forename of Cerulia’s foster father.

In Burney’s drama, the onomastic level is therefore essential, the true invisible engine of the dramatic action, with significances that would have been noticed by the contemporary public. A further example is the coryphaeus shepherdess Agatha, whose name – a straightforward sign of the Arcadian architext – has additional aesthetic and moral significance, as proven by a slightly later novel, Mary Wollstonecraft Shelley’s *Frankenstein, or The Modern Prometheus* (1818, second ed. 1831).

Agatha de Lacey, the daughter of the exiled M. de Lacey whom Victor Frankenstein’s Creature meets while rambling the woods, may in a sense embody the Creature’s first encounter with beauty.

I beheld a young creature, with a pail on her head, passing before my hovel. The girl was young and of gentle demeanour, unlike what I have since found cottagers and farm-house servants to be. ^26^

The name Agatha *–* feminine form of the Greek name ἀγαθός ( *agathos*), meaning *good*, *noble* – like those of the other actors in the sub-plot revolving around the de Laceys (Safie, Felix), with their symbolic Rousseauvian associations, must have been purposely chosen by Mary Shelley, as Anne K. Mellor pointed out many years ago: “As their symbolic names suggest, Felix embodies happiness, Agatha goodness. They are then joined by Safie ( *sophia* or wisdom).” ^27^

Symbolic onomastics informs and directs the dramatic plot at the deepest of levels. In the semi-historical universe of *Hubert de Vere –* the romanticized chronotope of the real Isle of Wight during the reign of the real King John *–* Cerulia is the only character to have a clearly unrealistic proper name, following a literary tradition well known to Burney. Her strategy is confirmed in those same months by the character sketches for the draft of her novel *Camilla* and, from a different angle, the formidable onomastic ordeal modelling the plot of her previous novel, *Cecilia*, centring on the (im)possibility for a wealthy heiress to keep both the surname she was born with and her inheritance after marriage.

A name can be a metonymy, and therefore a critical presentation, of a personage; it may be a foreboding, a proleptic sign of destiny, at times of doom. As a substantivation of the adjective ‘cerulean’, the name Cerulia transubstantiates a natural quality and a colour, usually associated with the sky or the sea, into the character of the predestined victim of fate and men *–* the *mulier necans* (dying woman) of the tragic tradition. As a nominal abstraction, “Cerulia” denies and deprives of identity and individuality its bearer. In fact, *Cerulia* is what I would call an *a-name*, in a construction combining the word *name* and the privative prefix *a-*: to me, Cerulia is *the denied-a-name*.

Where all the characters in Burney’s court tragedies have real or plausible proper names (Odo, Eltruda, Geoffry, the Earl of Chester), the name Cerulia stands out as a dis-embodied neutral quality or, rather, a sign of rhetorical figuration: it is an antonomasia. The maiden Cerulia is a helpless body deprived of an identity, in the catastrophic passage from her being dead to the world to truly dead: throughout, she plays a corpse-in-the-making. Not least, her character also seems to take on the auto-biografictive traits of the victimised Frances. At the time she was drafting the play, Frances the daughter was condemned to death-in-life by service to a sovereign from whom she could not free herself, torn as she was, in her very own she-tragedy, between the (strong) push of her socially-aspiring father Charles’s honour/service to the nation on the one hand and the (much weaker) pull of duty/love for herself and her own survival on the other.

Cerulia’s a-identity is confirmed, on the symbolic level, by the rich network of natural signs used to polarise the characters in the play through terms of comparison referring to the semantic fields of flora and fauna. While Hubert de Vere is compared, for example, to a “Red-breast” and a “Dove” (likely, *the* divine dove), similarity demotes Cerulia to a delicate “turtle Dove.” At one extreme, Cerulia is fragrant “Hawthorn”, a delicate “Rose”, a shy “Violet” growing in the shade, as if she embodied the very spirit of nature. De Vere, on the other hand, is reminiscent of a solid, imposing (and to me, somewhat grotesquely phallic) “Beech” tree at the other. ^28^

The list of these natural polarisations could go on (and it would be interesting to explore these significances further), but I choose to draw attention to the simile that in my opinion substantially recodes *–* and in a way totally debunks *–* the pastoral genre as Burney and her contemporaries knew it. I am thinking of the disturbing *–* and ominous – comparison, advanced by De Mowbray, of De Vere with a “fierce Wolf” and of Cerulia with “a tender Lamb”: “How did this tender Lamb tame the fierce Wolf?” (III, p. 120). A comparison, this one, certainly not reassuring (a paschal lamb?), harking back to the archetypal animal sacrifice present in Greek tragedies, including Euripides’ *Iphigenia in Tauris*, creating a disturbing continuum between sacrificial animals and human victims. ^29^ After all, Greek tragedy’s ritual killings represent an act suspended between “heroic oblation” and “political crime”, in Anna Beltrametti’s potent definition, which also fits *Hubert de Vere* quite well. ^30^

*Hubert de Vere* as a ritual sacrifice as much as a tragedy of innocence, then. Shadows and survivals of cults and violent rites of the origins that seem to belong to a distant elsewhere, reworked on the stage into mythical or quasi-mythical fables. These are other places that are displaced and made distant, like the space-time of historical drama or in the popular Romantic oriental plays (or, for that matter, in the contemporary Gothic novel). The reification of Cerulia *–* the helpless and obedient victim of historical machinations, family politics, and gender conventions *–* is signalled by Burney’s strategy of a-nomination, which de-humanises the girl, making her part of the natural world destined to be overpowered by patriarchal history and culture, and against which tradition, convention, and social apparatuses fatally and inevitably clash.

The proper name Cerulia is a signified weighted down by the social and symbolic connotations of the signifier. It is the innocence theorised by Northrop Frye overwhelmed by what, in a paraphrase of his discourse, we may call (in)experience. ^31^ And what remains in *Hubert de Vere*'s dramatic universe of Enlightenment sympathy, we may wonder, the all-important element in human nature that according to contemporary moral philosophy (and aesthetic theories) not only made society possible but also made it good? And what of the Aristotelian aesthetics of pity and terror? Whose plight and doom can the audience feel sympathy for: the vindicated ‘other’ woman, Geralda’s? or perhaps the wavering, dithering De Vere’s? or, finally, Cerulia’s, whose “murther” (V, p. 161) is made to seem so conveniently inevitable?

## *Et in arcadia ego*: indexing *Hubert de Vere*’s big “Oh!”

In closing these considerations, I move on to the very threshold of the Big “Oh!” that I have rather cryptically evoked in the title of my essay. The O to which I refer is, first and foremost, the Theatre, the quintessential ‘wooden O’ (from the celebrated Prologue in Shakespeare’s *Henry V*) whose round, globe-like shape – like the Shakespearean Globe – refers on a symbolic level to the unparalleled complexity of the *theatrum mundi*.

Can this cockpit hold
  The vasty fields of France? Or may we cram
  Within this wooden O the very casques
  That did affright the air at Agincourt?
                                                      (ll. 12–15)

But other circles, other gaping pits, other stagings of verbal and identity voids weld together in the topological and familial claustrophobia of the Isle of Wight, in that country graveyard where *Hubert de Vere*’s tragic climax is set, on the soddy banks of a hollow grave. In these lugubrious final scenes, Cerulia, cruelly abandoned by de Vere in Act IV to honour his previous, fallacious promise to Geralda, narrates the terrifying vision she had: Hubert de Vere’s spirit, clad in “white” (V, p. 149), appeared to her at dead of night. This “most affrighting vision” (p. 151) materialized inside a gloomy church at the stroke of midnight, in a scene of powerful pathos, not least because of the evocative deixis: “CERULIA: […] in the bowels of the Earth | *these feeble hands* must fashion my last home” (p. 150; the emphasis is mine). Let us imagine for a moment the possible staging of this scene, perhaps with Sarah Siddons (who played the leading character Elgiva in Burney's *Edwy and Elgiva*, DL 1795) in the role of Cerulia addressing the awful vision in a tormented, terrifying monologue. Siddons had created a greatly admired, awe-inspiring revision of the famous handwashing scene in Shakespeare’s *Macbeth* (V. i), which she played on the Drury Lane stage in a flowing white robe reminiscent of a shroud. ^32^ The powerful infra-theatrical overcoding offered by Siddons’s Lady Macbeth, one of the signature roles of the star actress, would have been instantly recognizable by the Drury Lane audience, closet players and theatre-loving readers alike. ^33^

In obeisance to the vision, Cerulia must dig her own grave with her own hands. This extreme, beyond-the-limits action is related to the shepherdesses rather than staged, as was the case with death scenes in Greek tragedies. The narrative of the forbidding injunction issued by the stern vision that orders Cerulia to bury herself – alive? dead? does it make a difference anyway? – in a ritualistic service all centred around the symbolism of the number three is extraordinary and, above all, unexpected.

CERULIA *Three* hideous Spectres that before me glar’d.
  And *thrice* the torch was quench’d, and all was dark,
    All deadly gloom: -- all sound, all sight denied:
    And *thrice*, by touch unseen, the taper blaz’d
    Furious afresh, wav'd by the vision gaunt;
    […]
*Thrice* There I knelt; *thrice* call’d on Hubert’s name –
                                                        (V, p. 150: the emphasis is mine)

Three is the fatal figure that dominates the plot of *Hubert de Vere* and connotes its last scenes. It is a number which has to be understood as two+one, the undividable sum of love plus death fatally binding, in accursed triangles, De Vere, Cerulia and De Mowbray on one side and De Vere, Cerulia and Geralda on the other. As a tragically mundane antithesis to the divine trinity, this fateful three, a number at once ritual and symbolic, is the sign of the tragic ordeal experienced by the characters.

The graveyard scenes in Act V, shocking and horrific as they are, transmodalize the disintegration of the illusory Arcadia serving as *Hubert de Vere*’s thematic and ideological backdrop. Like the story of the emperor’s new clothes, this episode represents the drama’s “writing degree zero”, the one place where masks are cast off for once and collective and individual responsibilities starkly surface. ^34^ Thus, in a merger between the biographical, the historical, and the poetic, *Hubert de Vere* stages the generic oscillations and disintegrations of Romantic drama as much as the ambiguities and opacity of familial and social structures and conventions. Such fluctuations correspond to Burney’s own oscillations and predicaments at the time, under the sway of conflicting impulses in her new married life on the one hand and, on the other, daughterly duty to her meddlesome father, Dr Charles Burney.

In *Hubert de Vere* we have a death, a forbidden identity, and a narrative that gets interrupted on the threshold of saying and being, an agency denied to the woman-girl of nature. Cerulia’s grave of the mind is a very powerful sign to me, as powerful as the exclamatory ‘Oh’ which closes a poem, significantly untitled, composed by William Wordsworth c.1798–9, around the same time as the *Hubert de Vere* revision for “fire-side” reading that Burney mentions in the letter to her father of January 1797 that I quoted earlier. Wordsworth's poem, which many of you will recall as one of the so-called ‘Lucy poems’, eventually appeared in Volume II of *Lyrical Ballads* (1800) and it is relevant in a different way to the questions I raised in relation to *Hubert the Vere*.

She dwelt among th’ untrodden ways
  Beside the springs of Dove,
  A Maid whom there were none to praise
  And very few to love:
  A Violet by a mossy stone
  Half hidden from the Eye!
  —Fair as a star, when only one
  Is shining in the sky.
  She liv’d unknown, and few could know
  When Lucy ceas’d to be;
  But she is in her Grave, and, Oh,
  The difference to me! ^35^

Wordsworth's “Maid” (l. 3), a flower, a fragile child of nature, now rests, one wants to believe serenely, in her grave – yet another empty space of signifying absence. Her death, ignored by most, seems to make a difference only to the poetic writer. Yet who is this poetic ‘I’, I wonder, the enunciative ghost animating the forcefully stressed phrase "to me!" (l. 11)? After the lines of almost pantheistic pathos dedicated to the maiden, who is the all-directing ‘I’ behind this closing syntagm, he who emphatically cuts off the stanza and in so doing swiftly redirects the bull's-eye on himself just before curtain drop? Was this poetic writer a metaphorical accomplice in the death of the girl, a ghostly subject/object who passed almost invisibly over the face of the earth and whose name, Lucy (from the Latin name *lux*, genitive *lucis*, meaning ‘light’), is another natural metonym, almost another a-name, just as Cerulia’s? A benevolent interpretation would recognise in the poetic writer a sympathetic ‘I’, in alignment with the Graveyard school of elegiac poetry of the eighteenth-century tradition I mentioned earlier, a fellow human being who commiserates with the gentle victim of an untimely death and suffers for her loss.

From this angle, I think of Cerulia’s engulfing, hollow country grave as an imaginary glyph. As a polysemic O (or “Oh”, in the Wordsworthian echo I perceive), it indexes Burney's neverstage, the elusive ghost stage that accompanied her throughout her life; the gaping grave where Cerulia will lie low, burying herself and her identity, and finally, Wordsworth’s own performative ‘Oh’, urging the reader to pause and pay heed. To me, this threefold O of the mind is a *Stolperstein*, a disturbing stumbling stone. It is a non-icon, a semiotic phantasm, a potent sign indexing the emptiness and absence that synthetize and transmode Cerulia’s ill stars as well as all the denials and self-denials experienced by several of Burney’s female characters, notably her disciplined young heroines, Cecilia and Camilla.

In conclusion, *Hubert de Vere*’s possible un-stageability and its actual unstaged-ness allow Burney to conceive – within a precise, and very complex, historical and autobiographical chronotope (at the time of the King’s illness and during her exhausting service at court as Second Keeper of the Robes to Queen Charlotte) – a ritual space of imagination/expression that welds the author's aesthetic project to the political one, her vision of the world and history on the one hand to the expressive, possibly counter-expressive, possibilities of the stage on the other. In *Hubert de Vere*'s dysphoric neo-Arcadia, the pastoral myth is debunked and revealed to be a cultural mythologem, while Cerulia’s story is exposed as the perennial return of a destiny of oppression and injustice for a victimised and injured young woman, who is first and foremost a daughter. It is no coincidence that Burney admired Ann Radcliffe, the “Great Enchantress” of end-of-century Gothic (in Thomas de Quincey’s famous definition). The dreadful grave that Cerulia is enjoined to dig by the ghastly graveyard vision is yet another nightmarish version, perhaps all the more traumatising because unstaged and hence left buried in the collective unconscious, of the claustrophobic spaces of the revolutionary 1790s Gothic – be they locked up chambers, dark dungeons, imaginary prisons or, indeed, country graves.

## Ethics and consent

Ethical approval and consent were not required

## Data Availability

The data for this article consists of bibliographic references, which are included in the footnotes.

## Footnotes

^1^ “Nella famiglia cosiddetta tradizionale i sentimenti sono vincolati ai ruoli, mentre nella queer family è esattamente il contrario: i ruoli sono maschere che i sentimenti indossano quando e se servono, altrimenti meglio mai.” While the translation is mine, the title of Murgia’s post was originally in English. By kind permission of the Author. I would like to thank Irene Pipiciello of the *S&P Literary – Sosia&Pistoia* literary agency for her assistance.

^2^ Here, I take semi-serious license, like that used by John Gay in the title of his celebrated afterpiece *The What D'Ye Call It*, DL 1715.

^3^ The concept of “cultural capital” comes from the works of Pierre Bourdieu, namely his essay “The Forms of Capital”, in John G. Richardson ed., *Handbook of Theory and Research for the Sociology of Education* (Westport, CT, Greenwood Press, 1986 [1983]), 241–258, and the later book, *On Television and Journalism* (London, Pluto, 1998).

^4^ Frances Burney, *The Court Journal and Letters of Frances Burney (1786–1791).* In 6 vols. General Editor Peter Sabor (Oxford, Oxford University Press, 2011–2019).

^5^ Miriam J. Berkowitz transcribed a manuscript copy of *Edwy and Elgiva* (Hamden, CT, Shoe String Press, 1957). The first annotated critical edition of Burney’s tragedies and comedies was prepared by Peter Sabor and his editorial team (Frances Burney, *The Complete Plays*, General Editor Peter Sabor, 2 vols, London, Pickering and Chatto) in 1995. *Hubert de Vere* is in vol. 2, *Tragedies*, pp. 91–164.

^6^ The first of such letters is in Frances Burney [Madame d’Arblay], *Diary and Letters of Madame d’Arblay. Edited by her Niece* [Charlotte Barrett]. Vol. IV *1778–79* (London, Henry Colburn, 1854). The quote is on p. 224.

^7^ Frances Burney, *The Journals and Letters of Fanny Burney (Madame d'Arblay)*. Vol. 1. Ed. Joyce Hemlow (Oxford, Oxford University Press, 1972), p. 74.

^8^ Here, I have summarised coeval criticism and some contemporary comments from Joyce Hemlow, *The History of Fanny Burney* (Oxford, Clarendon Press, 1958); Margaret Anne Doody, *Frances Burney: The Life in the Works* (New Brunswick, NJ, Rutgers University Press, 1988); Ellen Donkin, *Getting into the Act: Women Playwrights in London 1776–1829* (London-New York, Routledge, 1994); Barbara Darby, *Frances Burney Dramatist: Gender, Performance, and the Late Eighteenth-Century Stage* (Lexington, University Press of Kentucky, 1997); Jaqueline Pearson, “‘Crushing the Convent and the Dread Bastille’: Anglo-Saxons, Revolution, and Gender in Women’s Plays of the 1790s,” in Donald Scragg and Carole Weinberg (eds.), *Literary Appropriations of the Anglo-Saxons from the Thirteenth to the Twentieth Century* (Cambridge, Cambridge University Press, 2000), pp. 122–37. Doody and Darby were among the first scholars to address in depth Burney’s four comedies, a small dramatic corpus that, unlike Burney’s tragedies, has received growing attention from both critics and stage practitioners over the last thirty years.

^9^ Editorial introduction to *Hubert de Vere*, in Burney, *The Complete Plays*, Volume 2: *Tragedies*, pp. 93–95. All quotes come from this edition. The play has no scene division. Hereafter, all quotes will be in-text, with indication of the Act in Roman numerals, as per convention. For ease of reference, this will also be followed by the page number.

^10^ I make a case for Burney’s multi-facetedness in Francesca Saggini, “‘Obscure’ Be Still the Unsuccessful Muse’: Frances Burney and the Arts”, in Francesca Saggini (ed.) *Frances Burney and the Arts* (Palgrave Macmillan-Springer Nature, Cham (Switzerland), 2022), p. 3.

^11^ William Holden Hutton, *Burford Papers: Being Letters of Samuel Crisp to his Sister at Burford; and Other Studies of a Century (1745−1845)* (London, Constable, 1905), p. 74 (my emphasis).

^12^ Claire Harman, *Frances Burney: A Biography* (London, Flamingo-Harper Collins, 2000), p. 261.

^13^ Both quotes in Frances Burney, *The Journals and Letters of Fanny Burney (Madame d'Arblay)*. Vol. 3. Ed. Joyce Hemlow (Oxford, Oxford University Press, 1973), p. 258.

^14^ Burney, *The Journals and Letters*, Vol. 3, p. 119; the emphasis is in the original.

^15^ Burney, *The Journals and Letters*, Vol. 3, p. 113. The italics and font effects are in the original.

^16^ Crisp’s paternal(istic) injunction originally reads as follows: “—Do You remember about a Dozen Years ago, how You Used to dance Nancy Dawson on the Grass plot, with Your Cap on the Ground, & your long hair streaming down your Back, one shoe off, & throwing about your head like a mad thing?—now you are to dance Nancy Dawson with Fetters on—there is the difference yet there is certainly a nameless Grace & Charm in giving a loose to that Wildness & friskyness sometimes—.” Frances, Burney, *The Early Journals and Letters of Fanny Burney*. Vol. 3. Ed. Lars E. Troide and Stewart J. Cooke (Oxford, Oxford University Press and McGill-Queen's University Press, 1994), pp. 238–39. I reconstruct the composition history of *The Witlings* and offer some hypotheses about the suppression of the comedy jointly orchestrated by Dr Charles Burney and Samuel Crisp in Francesca Saggini, *Backstage in the Novel: Frances Burney and the Theater Arts* (Charlottesville, University of Virginia Press, 2012), pp. 90–132 (partic. on pp. 127–30).

^17^ Burney, *The Journals and Letters*, Vol. 3, p. 258.

^18^ “Can you forgive our modern ancient piece | Which brings no chorus, tho’ it comes from Greece.” The quotes is from the “Prologue”, written by Mr Lloyd and spoken by Mr Garrick, in John Delap, *Hecuba. A tragedy. As it is acted at the Theatre-Royal in Drury-Lane* (Dublin, printed for W. Smith, J. Exshaw, H. Saunders, W. Whitestone, W. Smith jun. J. Potts, and S. Watson, Booksellers, MDCCLXII. [1762]), p. 10, ll. 7–8. *Eighteenth Century Collections Online* (accessed June 16, 2023). URL: https://link-gale-com.ezproxy.is.ed.ac.uk/apps/doc/CW0113607878/ECCO?u=ed_itw&sid=primo&xid=32f6fdbc&pg=5.

^19^ For example, see the early *A Tour of the Isle of Wight; the Drawings Taken and Engraved by J. Hassell.* In 2 vols. London: John Jarvis, 1790.

^20^ Frances Burney, *The Early Journals and Letters of Fanny Burney*. Vol. 4. Ed. Betty Rizzo and Lars E. Troide (Oxford: Oxford UP and McGill-Queen's UP, 2003), p. 192. I thank Paula Stepankowsky for her astute observation on an early draft of this essay.

^21^ Richard Helmholz, “Pope Innocent III and the Annulment of Magna Carta,” *The Journal of Ecclesiastical History* 69, 1 (2018): 1–14.

^22^ “Before and during the War of Independence a number of American Patriots had used the example of the English barons using force to compel King John to accept the terms of Magna Carta to justify their own resort to arms against what they regarded as Britain’s oppressive and arbitrary policies adopted since the early 1760s” (Harry T. Dickinson, “Magna Carta in the American Revolution.” *Magna Carta: History, Context and Influence*, edited by Lawrence Goldman (London, University of London Press, 2018), pp. 79–100, on p. 99). See the bas relief in the bottom right panel of the bronze door of the Supreme Court of the United States building.

^23^ The cartoon is freely accessible in Wikimedia Commons at the above URL. Accessed on June 17, 2023.

^24^ For the literary reverberations of this Act see Cara Norris, “The Suspension of Habeas Corpus and Narrative Proliferation in Wordsworth’s *The Borderers*”, *European Romantic Review* 17, 2 (2006): 197–203.

^25^ To the typical Restoration heroic pair Love-Honour Janice Farrar Thaddeus prefers “duty and family”, slightly recasting a dramatic convention in a way that I endorse, as will be clear hereafter. Janice Farrar Thaddeus, *Frances Burney: A Literary Life* (New York, St Martin's Press; London, Macmillan, 2000), p. 105.

^26^ Mary Shelley, *Frankenstein, or The Modern Prometheus*, Vol. II, ch. 3. Here and elsewhere in this essay, I prefer a general bibliographic indication, rather than referring to a particular edition, to encourage more widespread consultation.

^27^ Anne K. Mellor, “The Female in *Frankenstein*”, in *Romanticism and Feminism*, ed. Anne K. Mellor (Bloomington, Indiana University Press, 1988), reprinted as “Possessing Nature: The Female in *Frankenstein*” in *The Norton Critical Edition of* Frankenstein, ed. John P. Hunter (New York, Norton, 1996), pp. 274–86; on p. 277.

^28^ All the references come from: I, p. 101; I, p. 103; II, p. 108.

^29^ The character of Iphigenia is the model for the tradition of tragic daughters who swamped the late eighteenth-century stage, epitomising female heroism, extreme selflessness, and almost unearthly filial devotion. For one, the bold Euphrasia, the heroine of Arthur Murphy’s *The Grecian Daughter* (1772), was a particular crowd-pleaser, and one of star actress Sarah Siddons’s favourite roles. For the fortunes of *Iphigenia* on the eighteenth-century and Romantic stage, see Edith Hall and Fiona Macintosh, *Greek Tragedy and the British Theatre 1660–1914* (Oxford, Oxford University Press, 2005), p. 32.

^30^ Anna Beltrametti, “Ifigenia e le altre. Archetipi greci del sacrificio femminile o degli incerti confini tra sacrificio, oblazione eroica e crimine politico nella cultura ateniese del V secolo”, *Storia delle donne* 4 (2008): 47–69.

^31^ Northrop Frye, *Anatomy of Criticism: Four Essays* (Princeton, Princeton University Press, 1957).

^32^ Sarah Siddons appeared for the first time as Lady Macbeth at Drury Lane on 2 February 1785. See the interpretation of the Shakespearean character she gives in her *Memoranda*, reprinted as Sarah Siddons, “Remarks on the Character of Lady Macbeth”, in Thomas Campbell, *Life of Mrs Siddons,* 2 vols. (London, Effingham, Wilson, 1834), II, pp. 10–39.

^33^ I explain the concepts of Romantic “inter-theatricality” and “inter-playfulness” as follows: “Acting, we may assume, was intertextual in the sense that it often borrowed from techniques used in the context of one given performance as well as at the infratheatrical and intertheatrical levels (respectively, *infra-playfulness* and *inter-playfulness*). Actors consciously or unconsciously borrowed and imitated from their individual acting techniques, by quoting from each other’s and as well as from standardized styles, particularly in signature roles.” Francesca Saggini, *The Gothic Novel and the Stage: Romantic Appropriations* (London-New York, Routledge, 2015), p. 74.

^34^ Roland Barthes, *Writing Degree Zero* [ *Le degré zéro de l'écriture*, 1953], trans. Annette Lavers and Colin Smith (New York, Hill and Wang, 1977).

^35^ Capitals and orthography as in William Wordsworth, and Samuel Taylor Coleridge, *Lyrical Ballads*, edited with introduction, notes and appendices by R.L. Brett and A.R. Jones; with a new introduction by Nicholas Roe (London, Taylor & Francis Group, 2005), p. 199. Accessed June 15, 2023. ProQuest Ebook Central. The poem appears with the title “Song.”

