# Supplementary material for: The wolf, the lamb, and the big “Oh!”: voids, (w)holes, and epitaphic emptiness in Frances Burney’s Hubert de Vere
Source: Open Res Eur. 2023 Sep 6;3:138. [Version 1] doi: 10.12688/openreseurope.16439.1 (PMC10594047; doi:10.12688/openreseurope.16439.1)
Supplement: Peer review history

# Reviewer report: Reviewer response for version 1

**DOI:** 10.21956/openreseurope.17744.r34974

**Published:** 2023-11-13

**Authors:** Lorna Clark

**Affiliations:** 1 Carleton University, Ottawa, Canada

This essay examines Frances Burney’s little-known play, *Hubert de Vere*, exploring its links with the genre of Romantic drama, and in particular the Gothic. The author finds Burney’s unstaged (and possibly unstageable) tragedy written in the 1790s helpful in illuminating Burney’s compositional practices and in evaluating her professionalism. Given that Burney’s tragedies have been largely ignored or scanted by critics, the essay makes a valuable contribution if only by virtue of its subject-matter, adding to our understanding of Burney’s writings and her views on gender and politics, and linking her with the Romantic stage and larger cultural influences.

 The analysis is divided into four sections, though some receive more energy and attention than others. First, the essay traces out the timeline of the composition of *Hubert de Vere *and draws its implications. Then it explores the significance of the geographical and historical setting for the (potential) contemporary audience. The focus in the second half on the naming of Cerulia and the importance of the final scene pushes into new territory and assimilates seemingly unrelated (and apparently anachronistic) literary works, such as Shelley’s *Frankenstein* and one of Wordsworth’s Lucy poems, forging interesting connections across the boundaries of strict chronology.

 The first drafting of the play (which was later revised) took place in 1790–91, the climax and culmination of Burney’s unhappy court years; the author glances at its publication history (left in manuscript until 1995) and sparse critical reception. After setting up an interpretive binary in which Burney can be seen either as an unserious gifted amateur or a discerning professional, well versed in stagecraft, the author argues that (ironically enough) the very lack of professional success of *Hubert de Vere* makes it ideally suited to reveal Burney’s techniques, poetic practices and cultural influences.

 The second section explores the setting on the Isle of Wight whose pastoral scenery suggests an English Arcadia while expressing a Romantic aesthetic: the juxtaposition of the picturesque and sublime in Cerulia’s nightmare-like vision of the mouldering church would have showcased the skills of the best scene-painter of the day. The historical period is the reign of King John whose signing of the Magna Carta in 1215 is seen as the safeguard of British liberties though with an overlay of feudal privilege. However, the action of the play belies the false promise of equality and depicts the crushing of an innocent female victim (evoking the archetypal image of animal or religious sacrifice). The depiction of the sufferings of the village maiden represents a powerful expression of Burney’s gender and family politics.   

 While promising an appraisal of the female characters in the play, the essay focuses mainly on one, Cerulia, whose “a-naming” as the abstract embodiment of a quality or colour (cerulean) essentially denies her an identity and de-humanises her. She is associated with the natural world (the blue of sea or sky) and especially with flowers, whose beauty and fragility seems fated to be crushed at the hands of the hero, who is likened to birds and trees, and is (once) described as a wolf to the heroine’s lamb. In this reading, the hero is seen as anti-hero, forming a part of the social system which inexorably crushes the heroine, yet such a view would seem to overlook the complexity of De Vere’s character, a Byronic figure who roams the play tortured by anguish and equally a victim of the machinations of others. Even after achieving the object of his desire, De Vere does not seems destined to happiness but will remain haunted by the memory of the tragic sacrifice of innocent love. The reading does not fully account for the testimony of other characters to his virtue, valour, and nobility, nor explain the author’s purpose in giving him an equally abstract name, associating him with the quality of truth, ‘De Vere’, ‘of verity’. Hints may be given, though, when the author emphasises the prevalence of the figure three dominating the plot of *Hubert de Vere* and points to the doomed triangle of Cerulia, Geralda and De Vere, all of whom are linked by suffering and could be considered the victims of oppression.

 The climax of the essay – and of the play –comes with “the big Oh!”, the stage in Shakespeare’s Globe theatre, figured as a kind of void or lack at the centre of the action. The terrifying nightmare vision of Cerulia being forced to dig her own grave and seek her own annihilation enacts this sense of absence at the core. At this point, the author incorporates Wordsworth’s poem commemorating a flower-like innocent, Lucy, whose death arouses in the speaker’s mind the unbearable ache of loss. With ‘O’ (the letter with the empty core), the author symbolises this central void, likening it to “Burney’s neverstage, the elusive ghost stage that accompanied her throughout her life” and in which she buried her own identity.

 This highly original and moving insight concludes the essay which amply fulfils the promise made at the outset, to explore the play, *Hubert de Vere*, in order to illuminate aspects of Burney’s work and in particular her representations of women and gender, finding at the core her depiction of violence and the suffering of victimhood. The pain of self-denial and inner conflict afflicts several of her heroines and, one suspects, Burney herself, and remains a critical crux, lying at the heart of feminist interpretations of her work. In this essay, the author convincingly links Burney to the tropes of Romantic drama and especially to the female Gothic of the 1790s.

Is the argument persuasive and supported by appropriate evidence?

Yes

Is the work clearly and cogently presented?

Yes

Is the topic of the essay discussed accurately in the context of the current literature?

Yes

Does the essay contribute to the cultural, historical, social understanding of the field?

Yes

Reviewer Expertise:

Early English women writers; Burney studies; eighteenth-century novel; scholarly editing; biography.

I confirm that I have read this submission and believe that I have an appropriate level of expertise to confirm that it is of an acceptable scientific standard.

# Reviewer report: Reviewer response for version 1

**DOI:** 10.21956/openreseurope.17744.r35259

**Published:** 2023-10-23

**Authors:** Mascha Hansen

**Affiliations:** 1 University of Greifswald, Greifswald, Mecklenburg-Vorpommern, Germany

A timely essay arguing for the importance of considering Burney’s tragedies, unpublished during her lifetime, as an integral part of her oeuvre. Her extended use of Gothic elements, and focus on gender concerns, link her work to the Romantic era in ways that her novels do not, as the essay proves. Burney’s use of the recent trend for the Gothic, especially in *Hubert de Vere*, is moreover explained as evidence of her business acumen, and the play’s setting on the Isle of Wight in the times of King John discussed as part of her general aesthetic considerations as well as socio-political concerns (and perhaps an attempt to lure the public’s patriotic delight with historical tales).

 While the abstract seemed clear enough to me, it does not elaborate on the full scope of the essay; only the first passage outlines the argument that this all but forgotten play is to be considered not merely as part of Burney’s oeuvre but as part of the Romantic stage at large. This should be mentioned in the abstract, I think, and perhaps be explained in a little more detail: How do the research questions tie in with this argument? Moreover, the question raised in the abstract, “Does attempting to define the nature of the *hamartia* of which Cerulia remains victim lead the “ideal” reader/viewer toward either fate/the gods or, rather, social apparatuses?” does not seem to get an answer in the essay, it seems to me.

 This essay reveals the author’s erudition in every passage, but some basic information should be added for readers less familiar with Burney, e.g. the fact that there are two versions (as outlined in the preface to Stewart Cooke/Peter Sabor’s edition). If the plays were only for private circulation, and not published, how come contemporary reviewers called them “wretched” – does this refer to *Edwy and Elgiva*? Some citation seems necessary here.

 The references to Greek drama need a bit more explanation, too, as Burney did not read Greek – so where did she take the character’s names from? Did her brother Charles perhaps help her with their meanings? Or did she rely on, or refer to, other contemporary plays or translations? Do we know anything about Burney reading Greek tragedy?

 The structure, too, might be outlined a little more at the very beginning of the essay, so as to prepare the reader for some of the arguments, or the thread of ideas. Thus, for instance, I did not fully understand the argument concerning the names of Cerulia and later Wordsworth’s Lucy as “a-naming” (or “a-identity”), and the reference to Agatha de Lacy seems a bit far-fetched, unless the idea is that Burney revised the play’s pastoral references after having read *Frankenstein*?

 As a further suggestion, the secondary literature on the play (Barbara Darby, Margaret Doody), could be given some more consideration, though none of it is recent. This essay will, I hope, spark further interest in Burney's plays, and her engagement with  Romantic aesthetic categories.

Is the argument persuasive and supported by appropriate evidence?

Yes

Is the work clearly and cogently presented?

Partly

Is the topic of the essay discussed accurately in the context of the current literature?

Yes

Does the essay contribute to the cultural, historical, social understanding of the field?

Yes

Reviewer Expertise:

I have written a monograph and various articles on Frances Burney, focussing on her novels rather than her plays.

I confirm that I have read this submission and believe that I have an appropriate level of expertise to confirm that it is of an acceptable scientific standard.

# Reviewer report: Reviewer response for version 1

**DOI:** 10.21956/openreseurope.17744.r35263

**Published:** 2023-10-20

**Authors:** Maria Grazia Dongu

**Affiliations:** 1 University of Cagliari, Cagliari, Sardinia, Italy

The essay reconsiders Francis Burney's place in her time, particularly her association with Romanticism. Hubert De Vere is chosen as a case study because of its mixed literary genre, its fragmentary nature, and its exploration of gender issues, as well as the formation and coherence of the body politic and agency.

 The author explicitly states the questions that triggered her research and analysis from the outset. The article achieves its primary goal of gathering data and textual evidence to address the research questions. Subsequently, a fragment, a "wretched play," emerges as an experimental venture representing the conflict between outdated and modern models. The play's reinterpretation of spatial dichotomies, such as Arcadia Felix and Gothic landscape, is especially captivating. Francesca Saggini convincingly identifies the adaptation of old strategies to express new cultural tensions in the eighteenth century, particularly those between the aristocracy and humble classes, male power and female subjection.

 One of the most exciting sections of this article is devoted to the analysis of the relationships established within the microcosm of the family. 

 The first sign of a peculiar connection between the characters is the symbolic onomastics of the name Cerulia, which suggests a virtue, a celestial colour, banished from the world, virtually and literally dead. Cerulia and De Vere are defined by adjectives and symbolic renames, which once again recall the reworking of long-used words to convey the artist's personal struggles and, above all, to fulfil the task of expressing the issues and tensions of the eighteenth century.

 The last part is the most challenging. The author juxtaposes one of the poems from Wordsworth's Lucy with Cerulia's death and her grave-digging, which takes place offstage. Both are sudden and disturbing negations of expected and final words, a suspension of communication that creates a void. In Hugh de Vere, this void is significant. Above all, it indicates the author's self-effacement, the self-containment of women who do not make themselves public. Saggini rightly situates the work and Fanny Burney within the complex self-representation of women novelists, which is so much a part of the general rearrangement of cultural values in the 18th century. I appreciate how this scholar reveals her role as an intertextual reader, linking seemingly distant texts.

Is the argument persuasive and supported by appropriate evidence?

Yes

Is the work clearly and cogently presented?

Yes

Is the topic of the essay discussed accurately in the context of the current literature?

Yes

Does the essay contribute to the cultural, historical, social understanding of the field?

Yes

Reviewer Expertise:

Elizabethan and Jacobean Theatre; Eighteenth-century studies; Travel Writing; Translation.

I confirm that I have read this submission and believe that I have an appropriate level of expertise to confirm that it is of an acceptable scientific standard.
